# MONTI: A Multi-Omics Non-negative Tensor Decomposition Framework for Gene-Level Integrative Analysis

**DOI:** 10.3389/fgene.2021.682841

**Published:** 2021-09-10

**Authors:** Inuk Jung, Minsu Kim, Sungmin Rhee, Sangsoo Lim, Sun Kim

**Affiliations:** ^1^Department of Computer Science and Engineering, Kyungpook National University, Daegu, South Korea; ^2^Computing and Computational Sciences Directorate, Oak Ridge National Laboratory, Oak Ridge, TN, United States; ^3^Department of Computer Science and Engineering, Seoul National University, Seoul, South Korea; ^4^Interdisciplinary Program in Bioinformatics, Seoul National University, Gwanak-Gu, Seoul, South Korea

**Keywords:** feature selection, tensor decomposition, cancer, multi-omics, integrative analysis

## Abstract

Multi-omics data is frequently measured to enrich the comprehension of biological mechanisms underlying certain phenotypes. However, due to the complex relations and high dimension of multi-omics data, it is difficult to associate omics features to certain biological traits of interest. For example, the clinically valuable breast cancer subtypes are well-defined at the molecular level, but are poorly classified using gene expression data. Here, we propose a multi-omics analysis method called MONTI (Multi-Omics Non-negative Tensor decomposition for Integrative analysis), which goal is to select multi-omics features that are able to represent trait specific characteristics. Here, we demonstrate the strength of multi-omics integrated analysis in terms of cancer subtyping. The multi-omics data are first integrated in a biologically meaningful manner to form a three dimensional tensor, which is then decomposed using a non-negative tensor decomposition method. From the result, MONTI selects highly informative subtype specific multi-omics features. MONTI was applied to three case studies of 597 breast cancer, 314 colon cancer, and 305 stomach cancer cohorts. For all the case studies, we found that the subtype classification accuracy significantly improved when utilizing all available multi-omics data. MONTI was able to detect subtype specific gene sets that showed to be strongly regulated by certain omics, from which correlation between omics types could be inferred. Furthermore, various clinical attributes of nine cancer types were analyzed using MONTI, which showed that some clinical attributes could be well explained using multi-omics data. We demonstrated that integrating multi-omics data in a gene centric manner improves detecting cancer subtype specific features and other clinical features, which may be used to further understand the molecular characteristics of interest. The software and data used in this study are available at: https://github.com/inukj/MONTI.

## 1. Introduction

**Figure 1:**
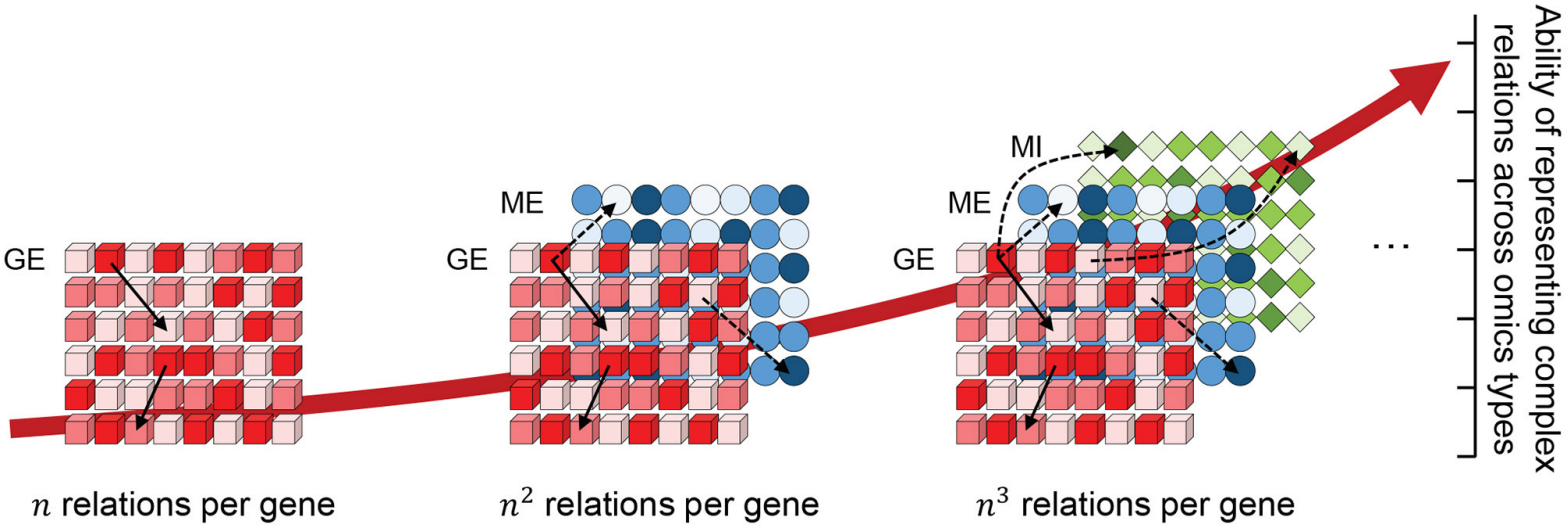
The possible number of relations that a gene can have across omics layers (GE, gene expression; ME, methylation; MI, miRNA) increases exponentially with each omics data type added to the integration. Here, *n* indicates the number of genes within a single omics layer.

Genes are among the most important building blocks of all organisms. Their transcription and translation are essential for maintaining fundamental cellular mechanisms. Genes are continuously and precisely regulated by a wide variety of mechanisms, including transcription factors, miRNAs, methylation, and mutations, which are often cumulatively referred to as multi-omics. When investigating a biological mechanism, each omics can only provide a single perspective. By matching multi-omics data sampled from a common subject, a multiple-perspective view can be generated for an enhanced understanding of the complex dynamics of biology in the subject. For each additionally integrated omics data type, a new relationship can be mined between a gene and the newly added, which increases the ability to represent complex relationships across multi-omics data types, as shown in Figure 1. However, due to their heterogeneous nature, it is difficult to integrate such different omics data types within a common data structure and even more difficult to analyze them in a combined manner due to their high dimension.

A number of initiative projects have made great effort to collect and publicly provide large amounts of multi-omics data, such as TCGA (Weinstein et al., 2013), GTEx (Carithers et al., 2015), ENCODE (The ENCODE Project Consortium, 2012), and HFGP (Li et al., 2016). These databases provide more than 10,000 high-throughput sequencing data sets generated using various platforms and collected from cancer patients, normal human tissues and model organisms. Compared to the availability of such large amounts of multi-omics data, the development of analytic methods that can encompass such large-scale heterogeneous data is just recently gaining interest (Hasin et al., 2017).

It is well understood that more data can improve the accuracy of data mining. However, this is true only if the data are precisely understood and, more importantly, correctly integrated. Omics data are generated on different platforms, which implies unique measurement scales, data formats, as well as different emphasis on molecular domains and relationships among molecular entities. Hence, normalization, pre-processing, as well as how to evaluate associations with genes or other entities must be carefully taken into account for each omics data set. Finally, the data must be analyzed in an integrative manner in order to data mine inter-relationships across the multi-omics domains.

While the aforementioned initiative projects are focused on providing large-scale multi-omics data, other databases have gathered and processed these large data sets to allow statistical queries. The LinkedOmics project (Vasaikar et al., 2017) collected multi-omics data from TCGA that includes 32 cancer types, surpassing 1 billion data points in total. Using simple correlation methods (i.e., Pearson, Spearman), a user may search for genes that are significantly correlated with the query gene. Here, the correlation is in the context of multi-omics. In addition to issues around data collection and analysis, methods for visualizing multi-omics data is important. With an increasing number of omics comes increased difficulty in visualizing the relationships between multiple omics. PaintOmics3 (Hernández-de Diego et al., 2018) is a web-based visualization tool that allows users to observe multi-omics relationships in a graphical manner. It supports nearly every sequencing technology platform, including proteomics and region-based omics data, such as ATAC (Buenrostro et al., 2015) or ChIP-seq (Park, 2009) data.

To date, studies sought to analyze high-throughput multi-omics sequencing data, with the majority reporting results using a single or a pair of omics (e.g, mRNA-miRNA, mRNA-methylation). In addition, the majority of such studies focus on identifying genes showing significant correlation with a certain omics type using statistical methods, such as Pearson's correlation or cosine similarity. Furthermore, such approaches tend to focus on finding a matching omics relation for a single gene with each iteration of the analysis rather than analyzing all genes and omics data in a combined manner. This is mainly due to the heavy computation load and requirements of multiple testing, which makes statistical analysis difficult.

A number of studies have reviewed multi-omics integration methods. A recent study (Huang et al., 2017) grouped multi-omics integration methods into four categories: (1) Matrix factorization methods, (2) Bayesian methods, (3) Network-based methods, and (4) Multiple step-analysis. In addition to those categories, the recently popular deep learning technique has been applied to predict genes that yield significant survival results in liver cancer (Chaudhary et al., 2017). Such multi-omics integration methods can also be categorized as supervised and unsupervised by making use of labels that represent the phenotype of the data, such as normal vs tumor sample. Tools such as jNMF (Zhang et al., 2012), MOFA (Argelaguet et al., 2018), and PARADIGM (Vaske et al., 2010) are unsupervised methods that mine gene clusters or modules associated with a phenotype of interest. Also, a network based multi-omics clustering method, SNF (Similarity Network Fusion) (Wang et al., 2014), was proposed that integrates multiple omics networks by weighted similarity of cluster samples.

**Figure 2:**
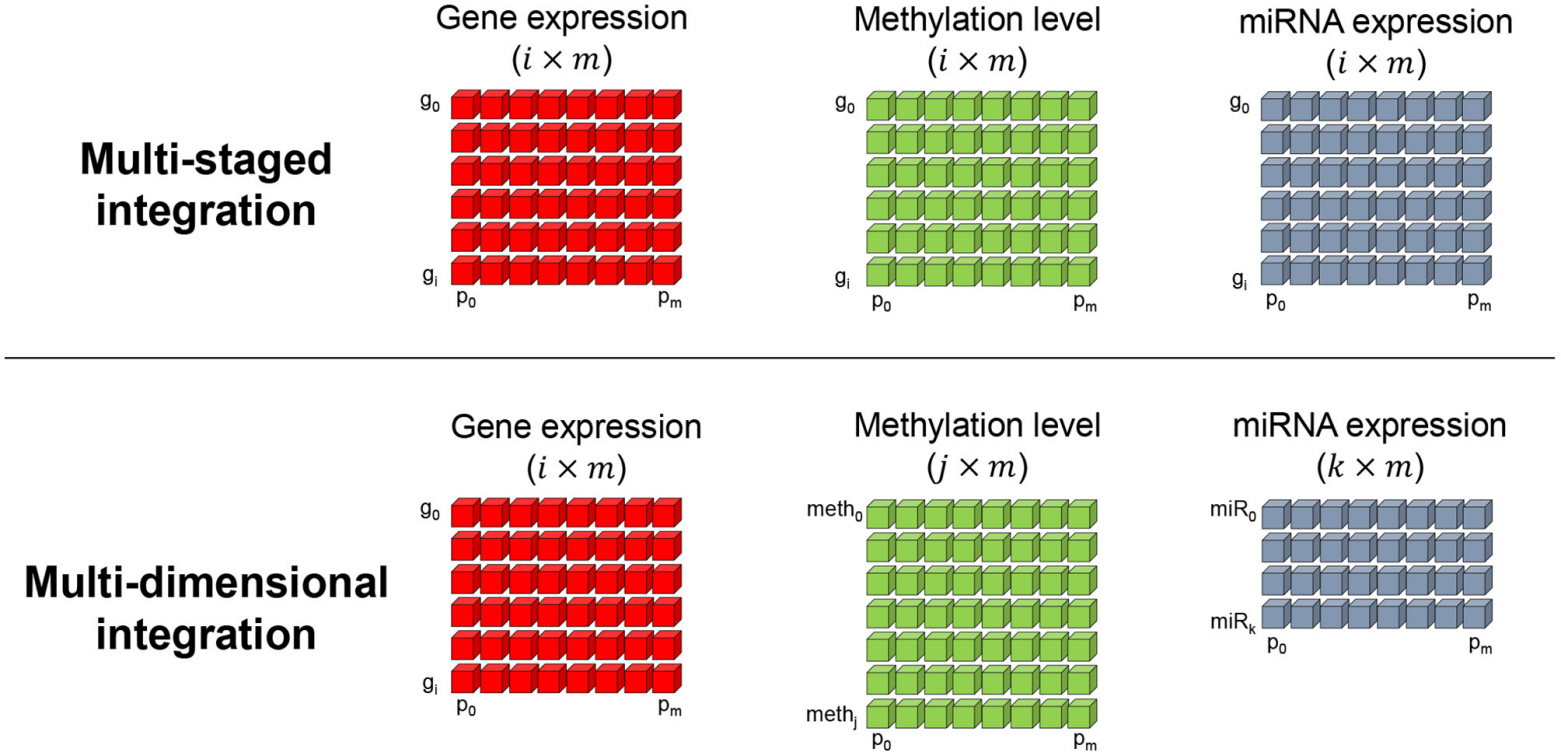
Two prevalently used multi-omics integration methods. The multi-staged **(top)**, or gene-centric, method encodes all omics measurement values in a per gene basis. Hence, the number of genes (*g*) and samples (or patients *p*) in each omics matrix are required to have equal dimensions. The multi-dimensional **(bottom)** integration method is less restrictive in the dimensions and makes use of each omics data as is.

More importantly, the aspect of the result greatly depends on how the multiple omics data are integrated. Two studies well-categorized and defined two important integration methods, which are the meta-dimensional and multi-staged integration approaches (Ritchie et al., 2015; Sathyanarayanan et al., 2020). The multi-staged integration method focuses on identifying omics factors that effect gene expression level, which is expected to find the causal relationship of a certain phenotype of interest. Hence, the omics data are integrated in a gene-centric manner and requires that each omics data have the same dimensions in sample and gene numbers as shown in Figure 2 (top). Here, *g* and *p* refers to the gene and patient (or sample) indices *i* and *m*, respectively. Such gene-level multi-omics integration can be advantageous in assessing the flow of information from omics to genes. For example, gene-level analysis of mRNA, methylation, and miRNA omics data can discover strong relationships across the three omics layers in means to explain the dynamics of gene expression (Subramanian et al., 2020). However, with limited number of omics data, the landscape of gene expression modulation may not be fully explained. Also, the selection of omics data need to be focused on the assumption that they influence the gene expression regulation. In the other hand, the multi-dimensional integration method makes us of each omics data as is. Thus, the number of entities in each omics matrix may differ. The two integration methods both assume a matched multi-omics, that is, multi-omics data are retrieved from the same subject and therefore have the same number of samples. Such assumption is also referred to as multi-modal data. Such omics-level integration may capture the bigger dynamics underlying a phenotype since the entire data is analyzed as is (Sathyanarayanan et al., 2020). However, to analyze relationships across the omics layers, post-processing of the result is required, which can become very complex with larger number of omics data since the combinations of omics exponentially increase.

Utilizing multi-omics data, we can identify important biomarkers and also identify multi-omics features specific to a given sample or phenotype. In the context of cancer, multi-omics features specific to cancer subtypes can be identified, which can serve as valuable information for constructing highly accurate subtype classification models. This approach will eventually facilitate enhanced identification of subtype-specific genes. Delineation between cancer and normal tissues or across different cancer types have long been a popular problem (Furey et al., 2000; Ramaswamy et al., 2001; Sotiriou et al., 2003), with a classification accuracy reaching 85% (Gevaert et al., 2006). However, classifying cancer subtypes (Network et al., 2012; Shen et al., 2012; Paquet and Hallett, 2015) is more difficult than distinguishing tumor and normal samples. For example, classification accuracy for predicting breast cancer subtypes is low, ranging from 56.7 to 75% (Wu et al., 2017; Tao et al., 2019).

In this study, we developed MONTI (Multi-Omics Non-negative Tensor Decomposition Integration) that learns hidden features through tensor decomposition for the integration of multi-omics data. MONTI is based on the gene-level integration method, which we find to be more helpful in understanding the results. The objective of MONTI is to extract feature genes that well explain some clinical attribute of interest in large multi-omics data. Being able to extract such a genes list with significant relation to clinical attributes can serve as a source that can naturally be used for simpler downstream analysis, such as, gene set enrichment of pathway analysis. Also, MONTI constraints the multi-omics data to be subject matched, where each omics data are collected from a common subject (i.e., patient). Such design may avoid omics variance within a same group, thus, amplifying the signals of hidden features.

In experiments with TCGA multi-omics data sets from breast, colon and stomach cancer samples, MONTI achieved significantly higher cancer subtype classification accuracy than existing multi-omics analysis methods. For the downstream analysis, genes associated with subtype-specific features were identified for biological interpretation.

## 2. Materials and Methods

### 2.1. MONTI Framework Overview

**Figure 3:**
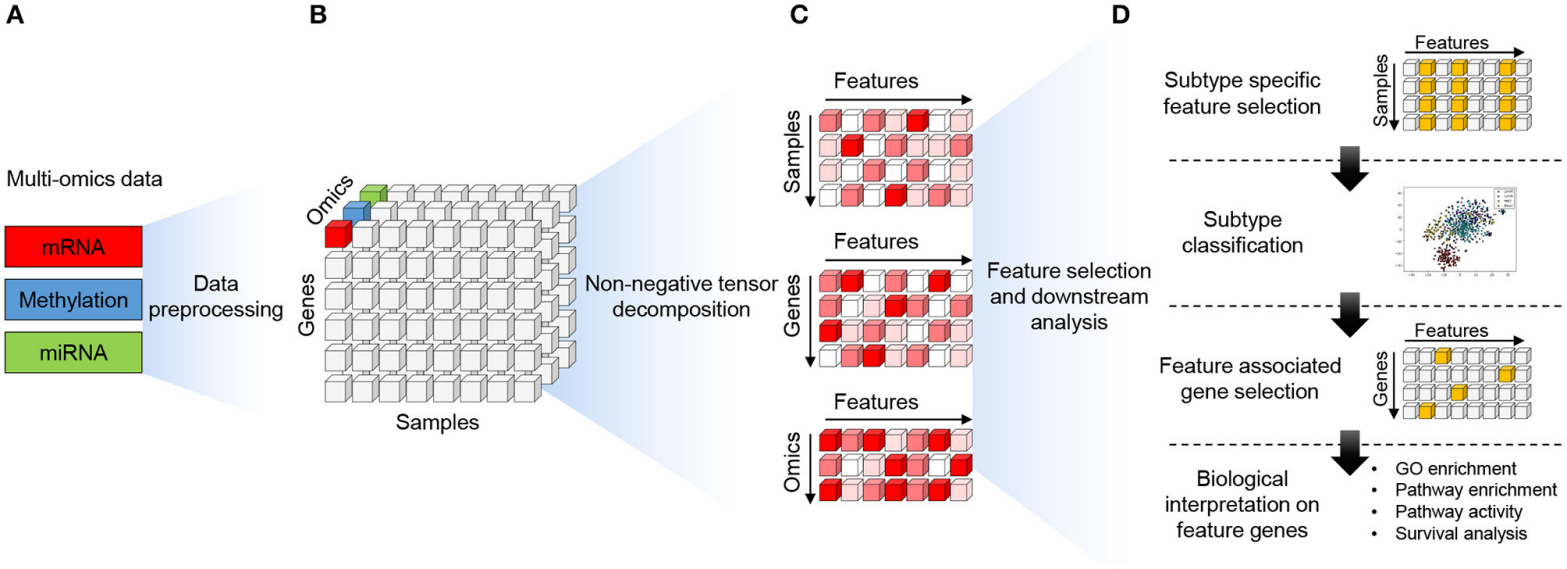
The workflow of the MONTI framework. **(A)** Each omics data (gene expression, methylation, miRNA expression) is pre-processed as a two-dimensional gene-centric matrix comprised of genes and samples. **(B)** The omics matrices are then stacked to form a three-dimensional tensor structure (genes, samples, omics) all sharing the same genes and samples. **(C)** Using the PARAFAC approach, the tensor is decomposed into two-dimensional gene, patient and omics components. Here, the components share the rank features. **(D)** The patient component is used to select subtype-specific features using subtype-specific L1 classifiers. The selected subtype-specific features are used to build a subtype classifier model using MLP (Multi-layer perceptron). Genes associated to the subtype-specific features are then selected for biological function analysis.

The MONTI workflow operates in two phases. In the first phase, the multi-omics data are integrated and decomposed using non-negative tensor decomposition. In the second phase, subtype-specific features and genes associated with them are selected using L1 regularization, and these features are then used to generate a subtype classifier using the multi-layer perceptron (MLP) neural network. The overall workflow is depicted in Figure 3.

### 2.2. Data Preparation and Preprocessing of Multi-Omics Data

Samples with matched gene expression, methylation, and miRNA expression data sets were collected for three case studies from TCGA: (1) 597 breast cancer samples, (2) 314 colon cancer, and (3) 305 stomach cancer samples. Only primary tumor samples with all three matching omics data sets were selected for the analysis. The pre-quantified gene and miRNA expression values from TCGA were used as provided. For the methylation data, we used the HumanMethylation450 BeadChip-based data and further selected probes located within the gene promoter regions (i.e., 2 Kb upstream of a gene's transcription start site). Subtype information were acquired from the original studies. The partially missing subtype information of the breast cancer case study was taken from Lim et al. (2018), which were generated by the PAM50 classification method (Parker et al., 2009). Sample case IDs and annotated cancer subtypes of the samples used in this study are in Supplementary Table 1.

Because we aim to discover gene regulatory multi-omics features, each omics data is individually processed to form a *gene-centric* two-dimensional sample(patient)-gene matrix. The values in each omics matrix are computed and assigned with respect to each gene. The tensor structure requires all slices to be of the same size. Thus, while each omics matrix is independently processed, they share the same set of genes and samples.

The gene expression values were preprocessed according to the provided TCGA level 3 gene expression data, which were subject to *log*_2_ quantile normalization across samples. For miRNA, they were first bundled per target gene, such that the number of bundles matched the number of genes. The geometric mean of miRNA expression per bundle was assigned to each corresponding gene. The expression values were then *log*_2_ quantile normalized. For methylation data, probes located within the transcription start site and 2 Kb upstream of gene promoter regions were grouped per gene. The average methylation level per gene was further quantile normalized.

Due to the nature of tensor decomposition, the omics value in each matrix need to be scaled within a common range. If not, an omics matrix with comparably large values, such as gene expression, would have a diminishing effect on other omics matrices with relatively lower values. Hence, normalized matrices are further scaled within the range of 0–1. Finally, the omics matrices were stacked on an orthogonal axis to form a three dimensional tensor structure.

### 2.3. Tensor Decomposition

There are several ways to decompose a tensor. PARAFAC (Carroll and Chang, 1970; Harshman, 1970) (a.k.a CANDECOMP-canonical decomposition) and TUCKER3 (Kroonenberg, 1983) are the most widely used methods. Both are multi- or bi-linear decomposition methods, which decompose the array into sets of scores and loadings. The decomposed scores and loadings describe the original data in a more compressed form. PARAFAC is based on factorization, whereas TUCKER3 utilizes principal component analysis. The resulting decomposition structure also differs between the two. PARAFAC decomposes a tensor into three two-dimensional components or matrices, while TUCKER3 generates three two-dimensional components along with an additional core matrix that is shared by the components. Due to the core matrix, interpreting data with the TUCKER3 model is more complicated (due to the increased number of parameters) than PARAFAC (Bro, 1997). Hence, here we used the PARAFAC method to decompose the multi-omics tensor.

A PARAFAC model of a three-way array *T* with elements *x*_*ijk*_ is given by three loading matrices, *C*_*g*_, *C*_*p*_, and *C*_*o*_ with elements *g*_*if*_, *p*_*jf*_, and *o*_*kf*_. Here, we refer to *C*_*g*_, *C*_*p*_, and *C*_*o*_ as the gene, patient and omics components, respectively. The tensor *T* is decomposed using a predefined number of ranks *R*, which we will refer to as features *f* = 1, …, *R*.

Due to the non-negative constraint, the interpretation of the feature values are much easier, since they are cumulative and do not negate themselves. Thus, a larger value will imply a strong signal of the feature. Furthermore, since omics data are most non-negative, the non-negative constraint can be naturally applied.

The trilinear model minimizes the sum of squares of the residuals, *e*_*ijk*_ in the model


(1) $$\begin{array}{l} x_{ijk}=\overset{R}{\underset{f=1}{\sum }}g_{if}p_{jf}o_{kf}+e_{ijk}, \end{array}$$

which can also be written as


(2) $$\begin{array}{l} T=\overset{R}{\underset{f=1}{\sum }}g_{f}⊗p_{f}⊗o_{f} \end{array}$$

**Figure 4:**
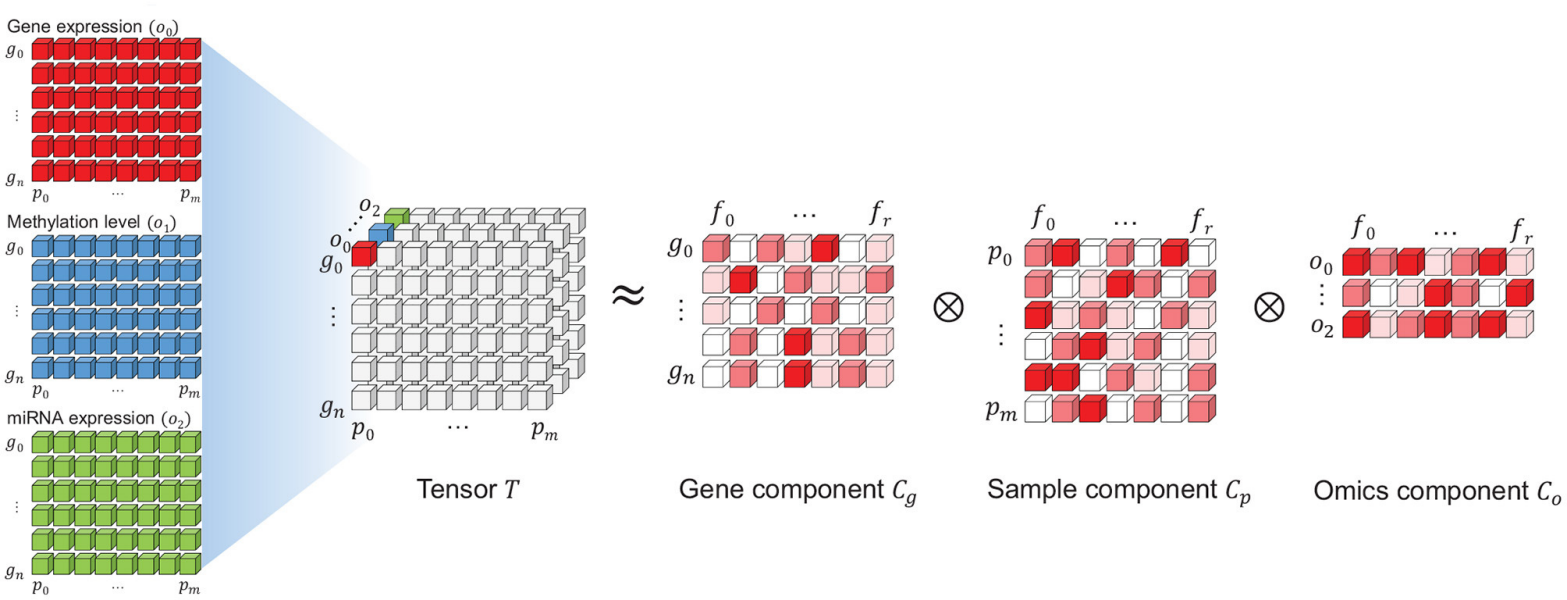
An illustration of the non-negative tensor decomposition (PARAFAC) using three types of omics. The tensor *T* is decomposed into three components: gene, sample (patient), and omics. Each component corresponds to one axis in tensor *T*. Each component is a two-dimensional matrix where one axis embeds the rank features *f*_*i*_(*i* = 0, …, *r*) for the entities in each component (i.e., genes, samples, and omics), similar to the traditional matrix factorization method.

An illustration of the PARAFAC model using gene expression, methylation level and miRNA expression data is in Figure 4. Here, *g*_*n*_(*n* = 0, …, *N*) refers to the genes, *o*_*k*_(*k* = 0, …, *K*) indicates the type of omics and *p*_*m*_(*m* = 0, …, *M*) refers to patient samples. *N*, *M* and *O* indicate the number of genes, samples, and omics types, respectively. Three omics types are used in this illustration; thus, *K* = 2.

### 2.4. Feature Selection

Subtype-associated tensor features, a subset of features selected from the tensor decomposition result, significantly improved subtype classification accuracy. To select such subtype-specific features, L1 regularization was used for each subtype and applied to the (*C*_*p*_) component (i.e., patient component) with the following equation,


(3) $$min\sum_{i=1}^{M}(y_{i}-\sum_{f=1}^{R}z_{if}w_{f}{)}^{2}+\alpha \sum_{f=1}^{R}\left|w_{f}\right|.$$

**Figure 5:**
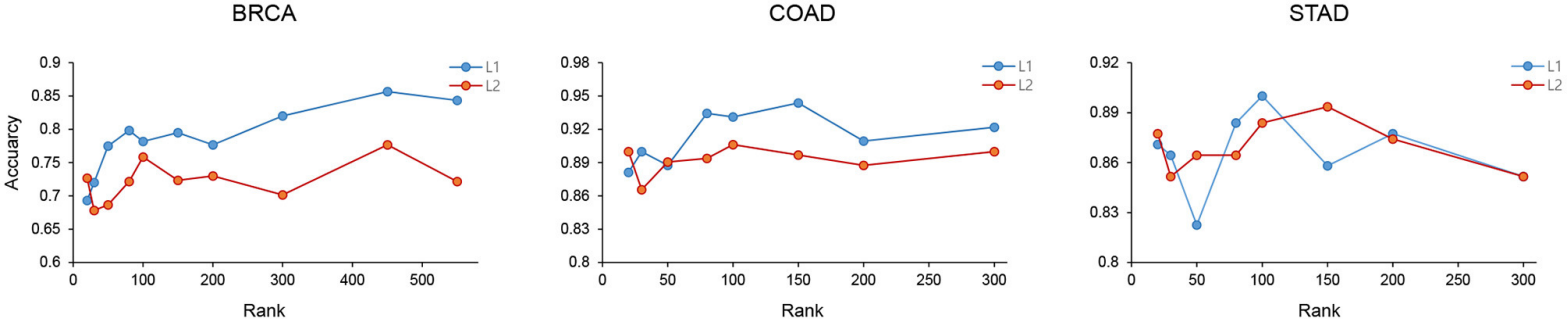
The cancer subtype classification accuracy of BRCA, COAD, and STAD was measured using features selected by the L1 and L2 method with different ranks.

Here, *M* refers to the number of patient samples and *R* the number of features, or columns, in *C*_*p*_. *y*_*i*_ refers to the target subtype value. Because an L1 model is built for each subtype, the target value is set to 1 for the corresponding subtype and 0 for the other subtype samples. For example, for the breast cancer case study, four L1 models were generated, one for each subtype of Luminal A, Luminal B, Her2, and Basal. *z* refers to the values of each feature in *C*_*p*_. *w*_*f*_(*f* = 1, …, *R*) refers to the weight of each feature to be inferred. The α value is the weight of the penalty term. Larger α values yields greater penalty, which will result in more features having zero weight and causing fewer features to be selected. We found that the L1 regularization achieved greater performance compared to the L2 regularization (Figure 5).

The feature selection performance using L1 and L2 were measured using the BRCA, COAD, and STAD data with varying ranks. As show in Figure 5, L1 showed better feature selection performance in terms of subtype classification accuracy in the three cancer types.

### 2.5. Selecting Feature Associated Genes

Based on the L1 selected features from *C*_*p*_, feature genes were further selected from *C*_*g*_. This procedure outputs a sparse set of genes, where each gene has a membership to a single feature. The association of a gene *g* to a feature is decided by *g*_*f*_ = *max*(*g*_0,*R*_), where the weight is maximum at the corresponding feature index *f*.

### 2.6. Cancer Subtype Classification Analysis

The significance of the selected feature genes was measured by their power of subtype classification accuracy. The classification accuracy was measured using a multi-layer perceptron (MLP) classifier with 10-fold cross validation. Here, values of the feature genes from *C*_*g*_ were given as input to build the MLP classifier.

## 3. Results

### 3.1. Three Case Studies

MONTI was applied to three cancer types: breast cancer (BRCA), colorectal cancer (COAD), and stomach cancer (STAD). The cancer types were chosen based on the number of samples that had matched multi-omics data from the same patient. There were 597, 314, and 305 matched omics data for BRCA, COAD, and STAD, respectively. To avoid an overly sparse tensor, genes that do not have any methylation probes located within their promoter and 2 Kb upstream of transcription start site (TSS) regions were discarded, which resulted in 14,513 genes with 60,707 methylation probes in total. The average methylation beta values were taken and assigned per gene. Similarly, miRNA expression values were grouped per target gene and the arithmetic mean of miRNA expression values in a group was assigned to its target gene. The multi-omics data items were used to produce gene centric omics matrices, which were then combined to form a three dimensional tensor of each cancer type, i.e., genes × multi-omics × patient samples.

### 3.2. Subtype Classification Results

Before deriving cancer subtype-specific features through tensor decomposition, a pre-defined rank *R* value for decomposing the tensor were needed to be chosen. In addition, a penalty strength, α value needed to be set for L1 regularization. Both were empirically chosen over a range of values by testing the subtype classification accuracy.

First, we evaluated the subtype classification accuracy using the feature in *C*_*p*_ over different ranks. The subtype classification accuracy for BRCA, COAD, and STAD was the highest with ranks 450, 150, and 100, respectively. The α value for L1 regularization determines the strength of the penalty for the features. The larger the α is the smaller number of features and genes be selected. Subtype classification performance was further investigated using α values ranging from 0 to 0.1. To further select informative features, the non-zero weight features were ranked by their absolute coefficient value from which top 20% features were chosen.

**Figure 6:**
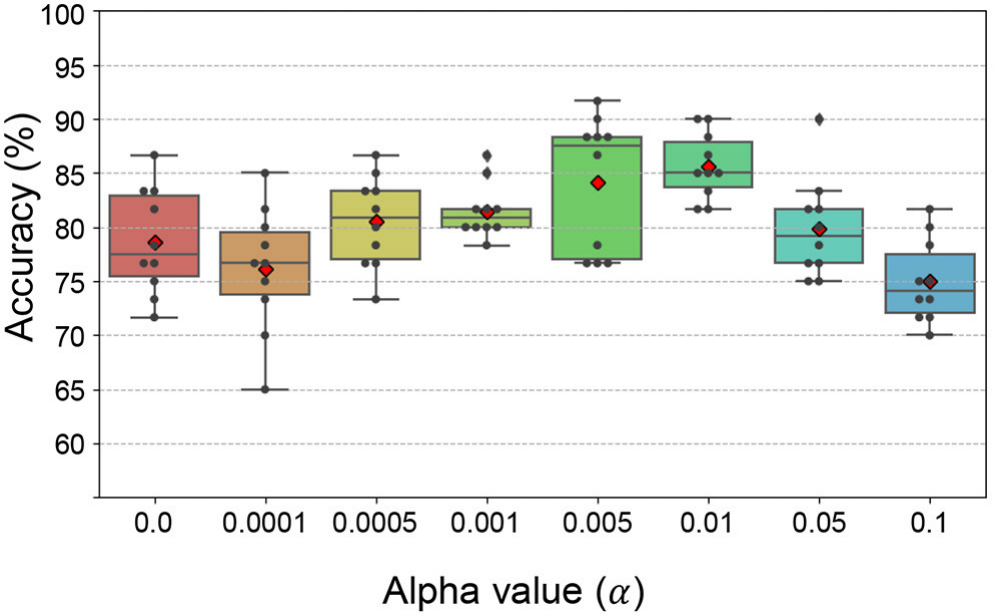
The classification accuracy with varying α values. The classification accuracy was the highest with α = 0.01.

The subtype classification accuracy was the highest when α = 0.01 (Figure 6). As a result, 26, 31, and 37 features from *C*_*p*_ were selected for subtype classification from the BRCA, COAD, and STAD tensors, respectively.

The multi-omics tensors for the three cancer case studies were decomposed with the optimal rank numbers and α values that were chosen as explained above. We then investigated how much contributions feature genes (i.e., from *C*_*g*_) made to the improvement in subtype classification accuracy.

**Figure 7:**
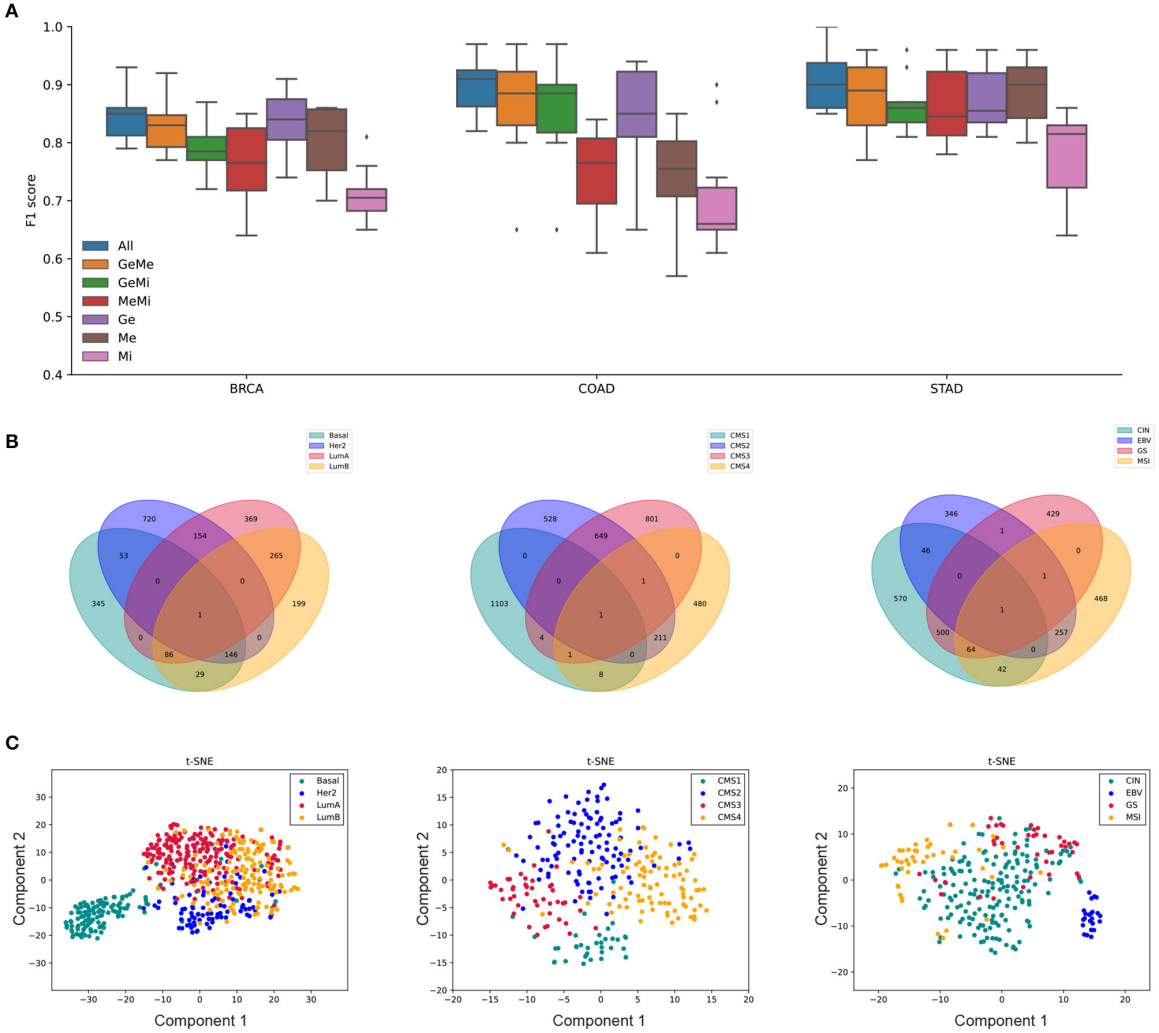
The MONTI analysis results of BRCA, COAD, and STAD subtypes are shown. **(A)** The subtype classification accuracy was the highest when using all three omics data for all three cancer types. **(B)** The cancer subtype specific genes. Here, the genes are shared by at most two different subtypes. **(C)** tSNE plots that were drawn using the selected features from *C*_*p*_ of each cancer type.

Our primary interest in this study was whether the selected features would better represent the underlying biological mechanism when using multiple omics data compared to single or a smaller subset of omics data. As shown in Figure 7A, subtype classification the accuracy was the highest when all available multi-omics data were used and combined by the tensor features, which are labeled as GE, ME, and MI for gene expression, methylation, and miRNA expression respectively.

Here, we find that such accuracy reflects how much the subtypes are explainable by the selected features and their associated genes in multi-omics manner.

The number of features and their associated genes are shown in Table 1. Since a feature can be associated with multiple subtypes, the sum of features in the St-Features column may be larger than the number of selected features. Here, Features and Genes refer to the total number of genes and the number of features in each cancer case study and St-Features and St-Genes to the number of genes and the number of features in each subtype St, respectively. A total of 2,385 genes, 3,831 genes, and 5,461 genes were found to be associated with BRCA, COAD, and STAD subtypes, respectively. The majority of genes were exclusively assigned to a certain subtype in all three cancer data sets (Figure 7B). This was more intuitive in the tSNE plot in Figure 7C. While the number of features was the largest in BRCA, the total number of genes did not necessarily differ with the other cancer types.

**Table 1:** The number of selected features and genes in BRCA, COAD, and STAD.

**Case study**	**Ranks**	**Features**	**Genes**	**Subtypes**	**St-Features**	**St-Genes**
BRCA	120	26	2,385	Luminal A	10	879
				Luminal B	9	732
				Her2	11	1,080
				Basal	8	665
COAD	120	31	3,831	CMS1	7	1,129
				CMS2	9	1,403
				CMS3	11	1,473
				CMS4	10	704
STAD	120	37	5,461	CIN	9	1,234
				GS	9	1,007
				MSI	9	839
				EBV	8	652

The 10-fold cross validated F1 scores of MONTI were 0.844, 0.9, and 0.91 for BRCA, COAD, and STAD, respectively. As far as we are aware of, the classification accuracy are highest among classification results reported in the literature so far and, in our experiments, MONTI outperformed existing methods such as MOFA2, iCluster, and SNF. For BRCA and COAD, the classification accuracy increased significantly when at least two omics data were used involving gene expression omics (GE). Improvement in classification accuracy was dramatic for COAD where use of single omics resulted in poor performance. Interestingly, methylation showed to be more influential in STAD, where ME alone achieved high classification accuracy. The CpG island methylator phenotype (CIMP) information can be used to characterize distinct subtypes of gastric cancer well and it is known that specific methylation patterns and clinicopathological features are associated (Network et al., 2014; Tahara and Arisawa, 2015) with it. While the majority of feature genes were associated with a single subtype (Figure 7B), some had membership to multiple. For example, the Venn diagram of BRCA shows that Luminal A and Luminal B subtypes share 265 genes while Her2 and Basal shared 53, which is true in the biological concept. Luminal A and Luminal B are hormone-receptor positive subtypes whereas Her2 and Basal are hormone-receptor negative subtypes, which also reflects the aggressiveness of the cancer (i.e., hormone-receptor negative cancers grow faster). Such characteristics are well-observed in the tSNE plots in Figure 7C.

### 3.3. Performance Evaluation

While few tools are available for multi-omics analysis with the goal of classifying cancer subtypes, all such tools aim to discover genes that have a strong correlation with one or more omics. In other words, such relational information is expected to differ between the cancer subtypes, which information is used to build classifiers or to mine subtype-specific data on genes or features. We compared the BRCA, COAD, and STAD subtype classification accuracy of five methods, which are MONTI, SNF (Wang et al., 2014), MOFA2 (Multi-Omics Factor Analysis) (Argelaguet et al., 2020), iCluster (Shen et al., 2009), and PCA.

The three cancer data sets consist of four subtypes. In BRCA, the number of samples per subtype were 220, 152, 91, and 132 for Luminal A, Luminal B, Her2, and Basal, respectively. In COAD, the number of samples per subtype are 43, 125, 48, 99 for CMS1, CMS2, CMS3, and CMS4, respectively. In STAD, the number of samples per subtype are 188, 26, 42, and 49 for CIN, EBV, GS, and MSI, respectively.

The genes used for analysis were chosen by two criteria. First, only protein coding genes were selected. Second, genes where the methylation values in the TSS 2 k upstream region was missing in more than 80% of the samples were filtered out. The miRNA data was used as is and the target gene information was acquired from mirDB (Chen and Wang, 2020). As a result, 14,514 genes were selected based on the BRCA, COAD, and STAD data sets. Methylation probes with missing values in all samples were dropped, resulting in 62,070 probes. Similarly, miRNAs with zero expression in all samples were excluded, resulting in 1,882 miRNAs. Each omics data were normalized as described in section 2.

**Figure 8:**
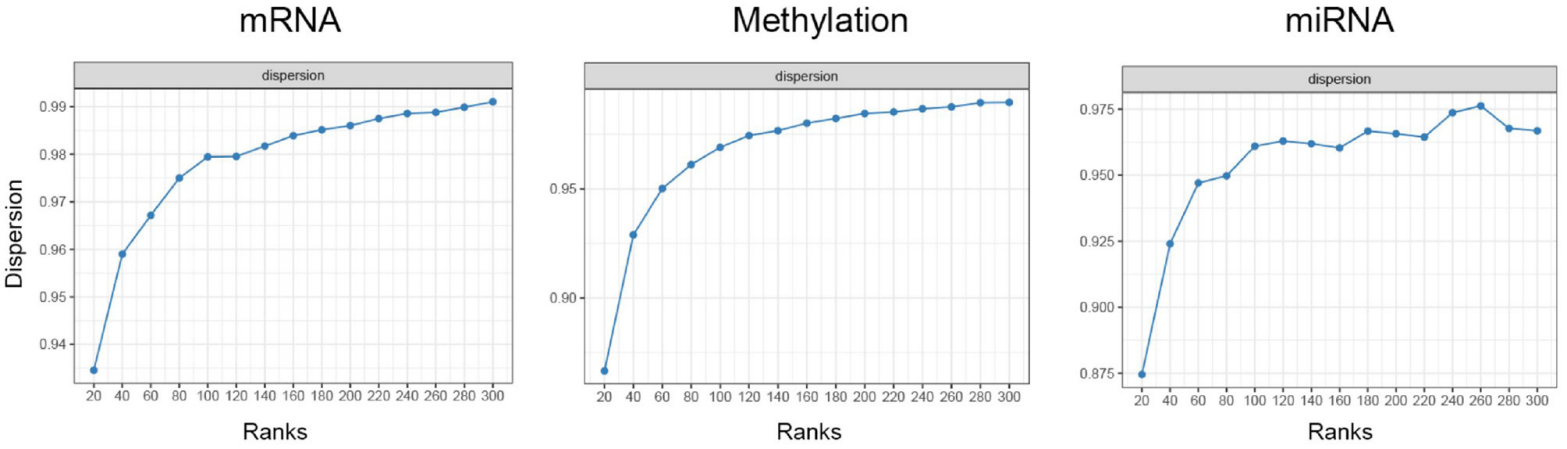
The dispersion plot using different ranks using BRCA omics data for estimating optimal NMF ranks.

The optimal number of ranks for MONTI were selected using the nmfEstimateRank function in the R preprocessCore package. For each gene-level omics data the optimal number of ranks were investigated based on the dispersion metric, from which we chose an appropriate rank number based on the elbow method. As a result, 120 ranks were chosen for BRCA, COAD and STAD. As an example, the dispersion plot of BRCA omics data are shown in Figure 8. The feature genes omics values were used for measuring the F1 score.

SNF (Similarity Network Fusion) integrates multi-omics data by constructing networks for each omics data in terms of the sample similarity using the omics data and then fusing the networks iteratively using the message-passing method. The principle is to keep edges between samples that are consistent across the different omics networks and to remove that are inconsistent and of low similarity. The optimal hyper parameters *K*, the number of neighbors in K-nearest neighbor, and *T*, the number of iterations for the diffusion process, where determined via the parameter grid search. The (*K*, *T*) parameters were set as (10, 30), (10, 10), and (5, 20) for BRCA, COAD, and STAD data sets, respectively. The output of SNF is the sample clusters, which was used to measure the F1 score.

MOFA2 utilizes matrix decomposition with the purpose of identifying sources of heterogeneity in multi-omics data sets. It decomposes multiple two-dimensional matrices, where each matrix represents an omics data type comprised of genes and samples. The decomposition yields feature matrices, each associated to one of the input omics matrices, and an additional factor matrix, which represents the activation values of each feature per sample. Thus, if three omics data are given as input, they will be decomposed into four matrices (i.e., three feature and one factor matrices). MOFA2 allows to chose the number of factors or features from the decomposed factor matrix, where we utilized as many as possible for each dataset. The maximum features that could be used was 10 for BRCA, COAD, and STAD, respectively. The output of MOFA was the Z sample factor matrix, which was used for measuring the F1 score.

iCluster adopts a joint latent variable model for integrative clustering of multi-omics data. iCluster aims to data mine significant associations between different omics data types through likelihood-inference using the Expectation-Maximization algorithm. iCluster supports a omics optimal weight estimation function, which we used for each data set for clustering. The output of iCluster is the sample clusters, which was used to measure the F1 score.

At last, sample PCA features were extracted and used for classifying the cancer subtypes. For each cancer and omics data, optimal number of PCA features were selected based on the classification accuracy via a parameter grid search. For BRCA, 10, 6, and 10 PCs were selected from gene, methylation, and miRNA data, respectively. Similarly, 8, 5, and 2 PCs for COAD and 20, 2, and 18 PCs for STAD were selected from gene, methylation, and miRNA data, respectively. The selected PCs were stacked and given as input to the random forest classifier to measure the F1 score.

The average F1 score was measured via 10-cross validation for each tool with configurations described above. The train and test data were split before any normalization or feature selection in each BRCA, COAD, and STAD data set. The same train and test data sets were used to measure the F1 score in each method. Furthermore, the input data were both prepared in gene-level (i.e., multi-staged) and omics-level (i.e., multi-dimension) format to observe the difference between the two integration methods. Thus, each method, except MONTI, was subject to two types of input data and were tested for classification accuracy accordingly. The tools measured with gene-level input data are labeled as SNF_g, MOFA2_g, iCluster_g, and PCA_g.

**Figure 9:**
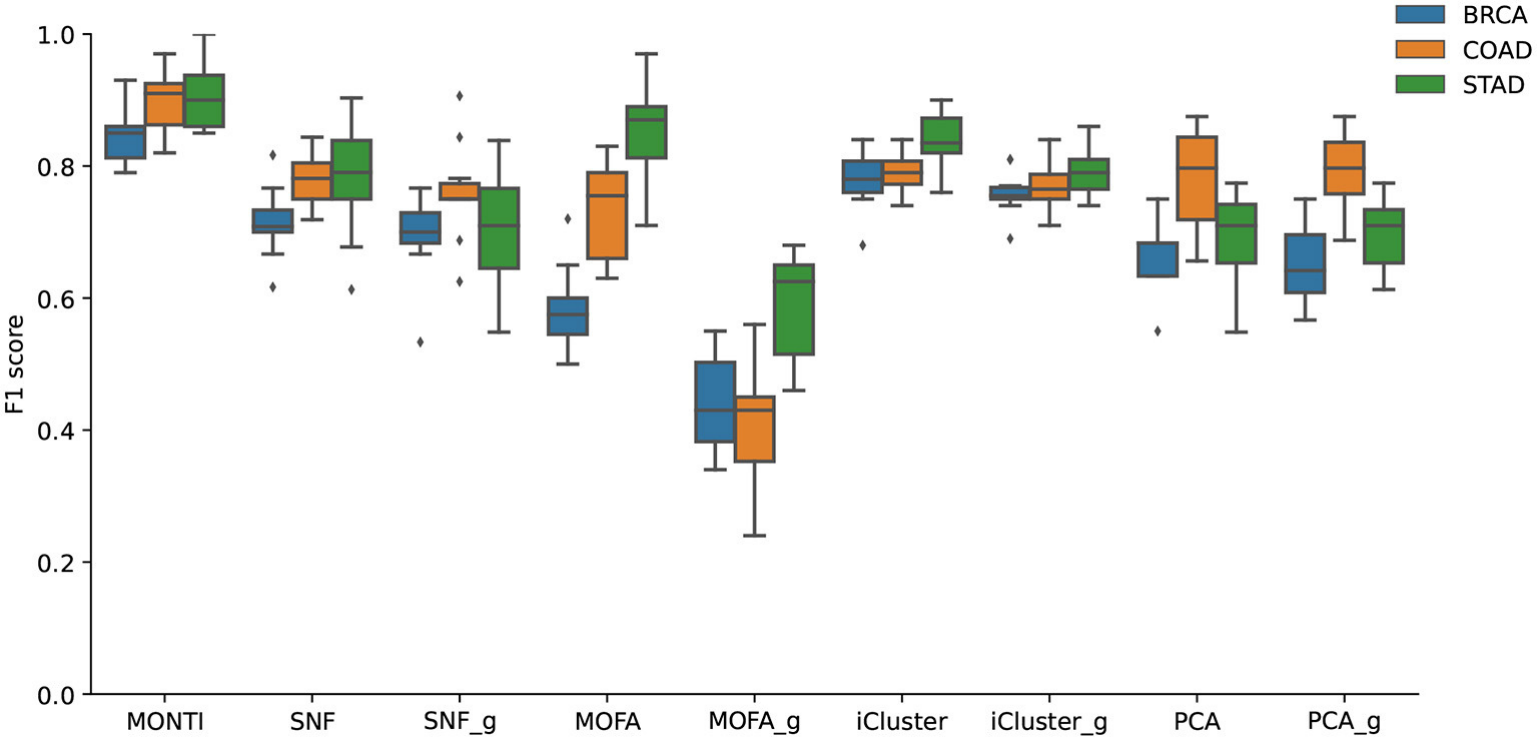
The F1 scores of five tools using gene-level and omics-level data sets of BRCA, COAD, and STAD subtypes.

The comparison results are shown in Figure 9. The F1 score was the highest in MONTI for all cancer subtypes, followed by iCluster and SNF. We observed that the gene-level input data yielded lower F1 scores in MOFA2, while it remained relatively similar in SNF, iCluster, and PCA methods. The significant drop of F1 score in MOFA2_g may be due to its feature extraction method. While the omics-level input data matrix is very dense, the gene-level matrix is relatively sparse, especially for the miRNA data. Hence, the latent factors associated with the miRNA data will loose information. Furthermore, while MONTI utilizes larger number of rank features, MOFA2 utilized 10 features, which may have reduced the dimension too much, thus, loosing more information accordingly.

### 3.4. Analysis of Pan-Cancer Clinical Features

The relatively high classification accuracy of the cancer subtypes above implies that they may be explained using the feature extracted genes in terms of multi-omics. Thus, we further investigated whether clinical attributes, other than cancer subtypes, such as gender, mutation groups or metastasis can be explained using multi-omics data. Among the many clinical attributes, categorical attributes with <5 groups were used. Also, clinical attributes with high sample bias were excluded. As a result, a total of nine cancer types and 95 clinical attributes were analyzed using mRNA, methylation and miRNA data. For example, the “Pathologic M” feature of STAD, which is the TNM staging of metastasis, has three classes, which are M0, M1, and MX. If the cancer has spread, the sample is labeled as M0, and if not it is labeled as M1. If metastasis cannot be measured, it is labeled as MX. Thus, similar to the cancer subtype classification, we measured the classification accuracy of each of the categorical clinical attributes that were selected by the criteria described above. The details of the data set and clinical attributes are provided in Supplementary Table 2.

**Figure 10:**
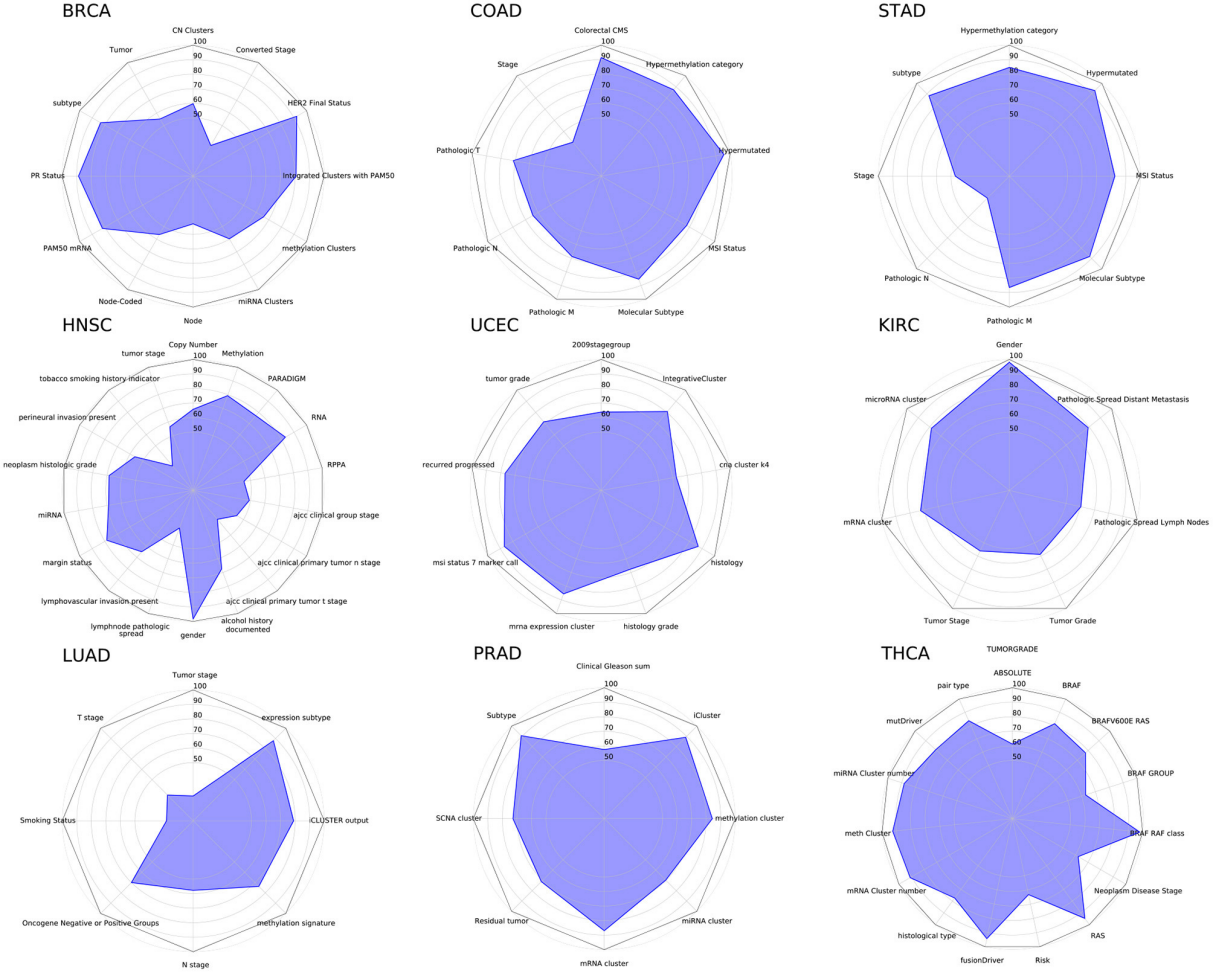
The radar chart showing classification results of nine cancers and their clinical attributes.

MONTI was executed on each cancer type and each clinical feature as described in section 2. The classification accuracy of the cancer clinical attributes are shown in Figure 10. Here, we observed that some clinical attributes were well classified while others showed poor classification.

All cancer subtypes showed relatively high accuracy in BRCA, COAD, STAD, and PRAD (Prostate adenocarcinoma), which hints that the multi-omics profile is highly correlated with cancer molecular subtypes. Also, while mutation data was not utilized, the BRAF and RAS mutation classes were well distinguished in THCA (Thyroid carcinoma). From such result, we may infer that at least mRNA, methylation and miRNA omics have causal relationship with BRAF and RAS mutations, which was also reported in Agrawal et al. (2014). In case of HNSC (Head and Neck squamous cell carcinoma), the gender attribute was classified with almost perfect accuracy, which was also reported in Yuan et al. (2016).

The Pan-cancer analysis results show that some clinical attributes are able to be explained using mRNA, methylation and miRNA data while others need further investigation using other omics or clinical data. Collectively, we find that such results may help selecting omics when performing research on clinical features in a cancer cohort.

## 4. Discussion

While not shown in this study, the subtype classification accuracy decreased when involving certain omics types, particularly with the use of mutation profile data. For BRCA data, the accuracy dropped below 0.75 when SNP data were included in the tensor. The first short-coming of the SNP data was its extreme sparseness (i.e., 0.5% genes with SNP). We further attempted to impute the remaining missing values using the network-based stratification method for tumor mutations (Hofree et al., 2013). Unfortunately, the accuracy further decreased, which may be due to the introduction of additional uncertainty arising from large number of predictions. For sparse data, integration methods that are not gene-centric may be more advantageous, such as SNF. Such result implies that no single method may be universally applicable for incorporating all types of omics data, and that omics data must be well understood and integrated in a manner specific to the characteristics of each omics. Similar arguments have been discussed previously (Zhang et al., 2018).

**Figure 11:**
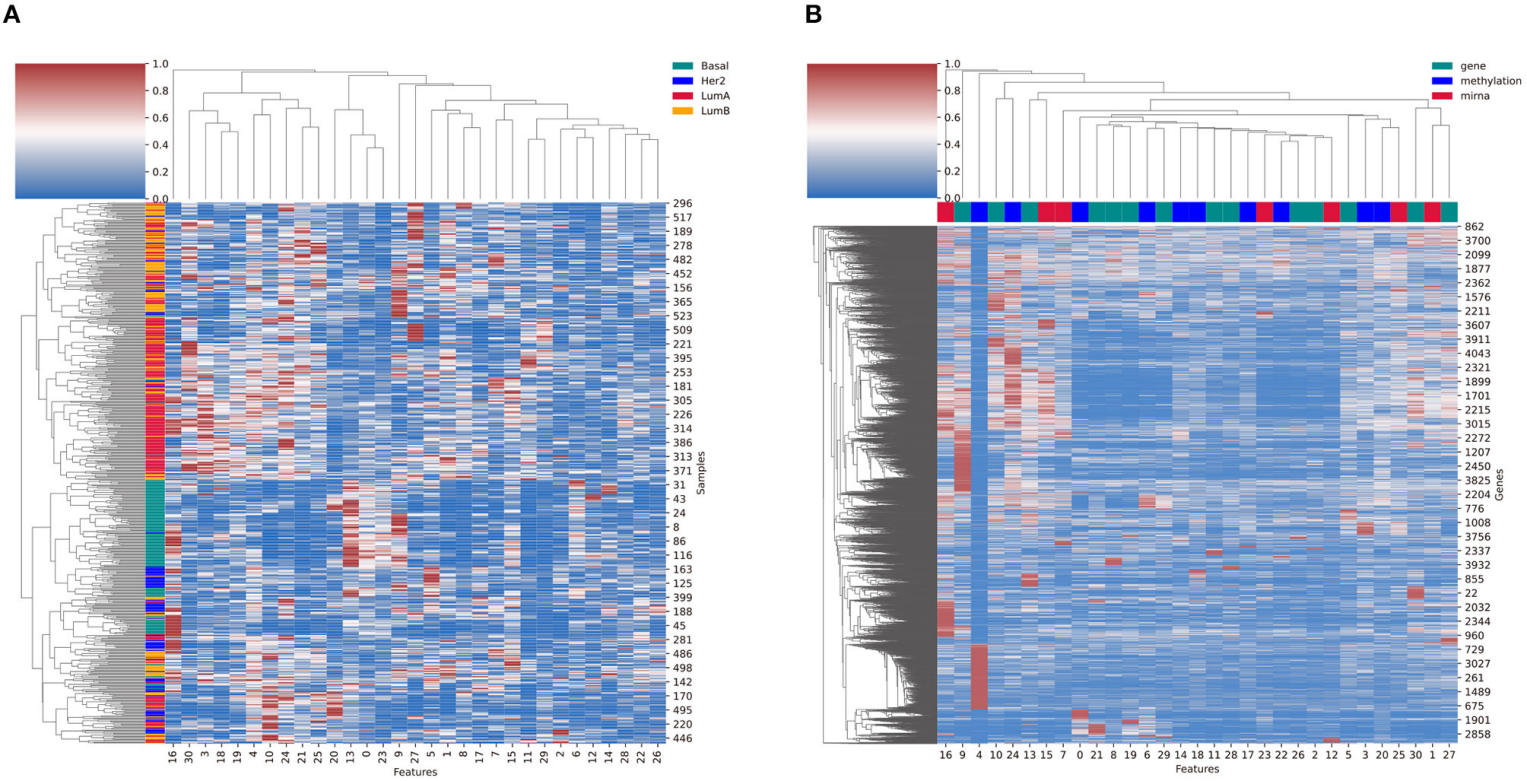
**(A)** The cluster heatmap of sample features (*C*_*p*_) and **(B)** the cluster heatmap of the feature genes (*C*_*g*_) from the breast cancer result. The left color bars in **(A)** refer to the BRCA subtypes. The top color bars in **(B)** refer to the omics with the largest feature value in *C*_*o*_.

Clustering of the selected sample features from the *C*_*p*_ component of the BRCA analysis result shows us that the Basal samples are well clustered together, whereas the Luminal A and Luminal B subtypes are relatively more mixed (Figure 11A). Similarly, the clustering of selected feature genes from the *C*_*g*_ component showed the feature activity of genes (Figure 11B). Here, the top color bars represent the maximum omics type of each feature. The feature four related genes had strong relation with methylation. Genes with high values in multiple features that are related with different omics types indicate that the gene has relationship across the two different omics types.

**Figure 12:**
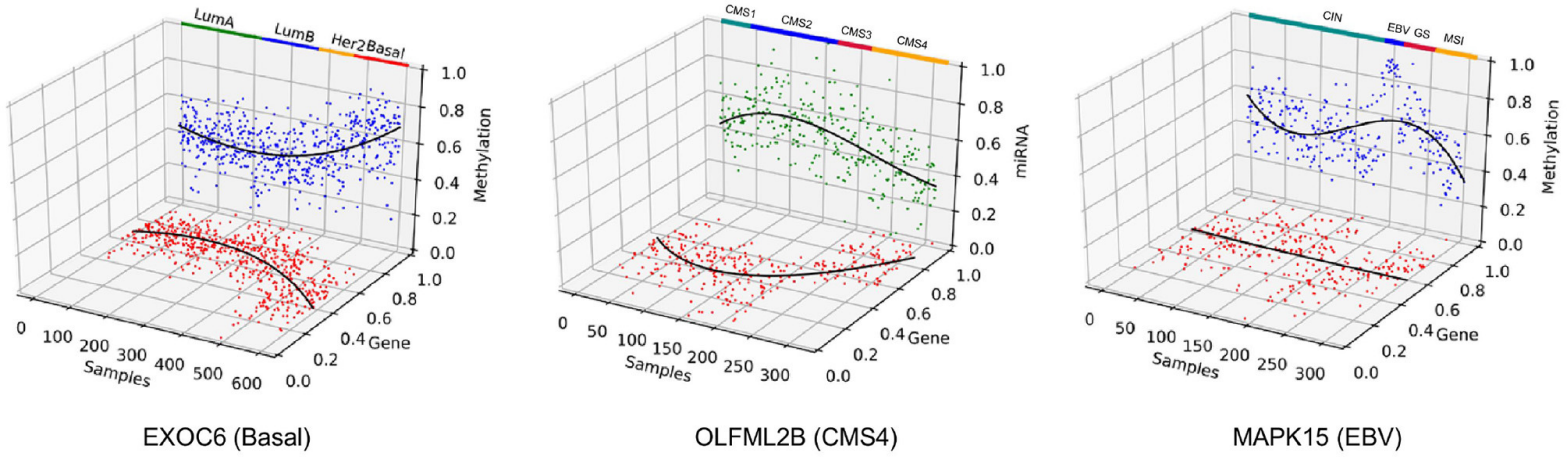
Three genes were selected to show correlation between different types of omics data across patient samples. EXOC6 was associated with the Basal subtype of BRCA, OLFML2B was associated with CMS4 subtype of COAD and MAPK15 was associated with the EBV subtype of STAD.

Furthermore, the selected features in all three case studies captured correlation among different omics data types. As shown in Figure 12, EXOC6 was most affected by DNA methylation in Basal subtype of BRCA. EXOC6 is reported to be an important respondent gene when the effects of a combination of the histone deacetylase inhibitor suberoylanilide hydroxamic acid (SAHA) and taxanes were tested for cytotoxicity using human breast cancer cell lines (Chang et al., 2011). Also, EXOC6 was found to be one out of five genes that was able to asses breast cancer risk with high accuracy (Winham et al., 2017). While EXOC6 was observed to have distinct methylation profiles in brain tissues (Farlik et al., 2016; Hira and Gillies, 2016), it was not actively investigated in breast cancer Basal subtype samples in terms of multi-omics correlation. The OLFML2B gene was found to be negatively correlated with miRNA in the CMS4 subtype in COAD. We found that the miRNA OLFML2B targeting miRNA, miR-30b, is a well-known oncogene suppressor miRNA in colorectal cancer (Liao et al., 2014), which may explain the omics relationship here. At last, the MAPK15 has been reported to be a regulator for redioresistance in nasopharyngeal carcinoma cells, which is tightly linked to the Epstein-Barr virus (EBV) infection (Li et al., 2018), which may relate to the EBV subtype of STAD. Collectively, we may induce that the MAPK15's expression is down-regulated by methylation, which was not the case in other STAD subtypes. Other than the selected genes, well known multi-omics correlated genes related to certain cancer subtypes were also detected. Although data not shown, the ESPL1, detected by MONTI, showed significant regulatory relationship between gene expression and methylation specific to Luminal A and Luminal B subtypes in BRCA, which was previously reported in Finetti et al. (2014) and Li and Li (2020).

OLFML2B was most affected by miRNA in CMS4 subtype of COAD. MAPK15 also showed strong gene expression regulation by methylation in EBV subtype of STAD. This kind of result by MONTI may suggest cancer subtype specific gene regulation mechanisms, which can help discover subtype-specific gene markers for further biological and clinical investigations.

The genes were further examined to see if they captured known signals of cancer subtype specific pathways by applying the Subsystem Activation Scoring (SAS) method (Lim et al., 2016). SAS is used to decompose molecular pathways into sub-pathways (named subsystems) and measure the activation levels of them in terms of gene expression. We expanded it to multi-omics levels to evaluate the association of each subsystem with each cancer subtype by constructing random forest classifiers using its SAS score. The detailed method and results are described in Supplementary Table 3. The detected pathway subsystems were highly specific to each cancer type. For example, the top 10 ranked pathways for the three case studies were all supported by previous studies. For example, the “Fanconi anemia” pathway was the top ranked pathway for the BRCA data, which is known to be a rare chromosomal instability disorder that is susceptible to cancer (Alan and D'Andrea, 2010). The “HIF-1 signaling” pathway was top ranked in STAD with association to miRNA. The study (He et al., 2017) suggests that miR-224 promotes cell growth migration and invasion by targeting the RASSF8 gene in STAD. Similarly, the top ranked “Vascular smooth muscle contraction” pathway by SAS was also reported to be induced by colorectal cancer (Li et al., 2017).

The application of MONTI was demonstrated on cancer subtype multi-omics data. However, MONTI is not tailored to cancer subtype analysis but can be utilized to identify any categorical clinical features, such as gender, mutation groups, tumor grade, or age. Thus, the advantage of MONTI is that it is able to identify clinical feature associated genes in terms of multi-omics. Furthermore, the omics component *C*_*o*_ can be further used to investigate which omics are currently active and take part in gene expression regulation.

## Data Availability Statement

Publicly available datasets were analyzed in this study. This data can be found at: TCGA multi-omics data.

## Author Contributions

SK and IJ designed the project and MONTI algorithm framework. IJ and SR implemented multi-omics integration. SK, IJ, SL, and MK performed the biological analysis and interpretation.

## Funding

This research was supported by the Bio & Medical Technology Development Program of the National Research Foundation (NRF) funded by the Korean government (MSIT) (2019M3E5D3073365), the Collaborative Genome Program for Fostering New Post-Genome Industry of the National Research Foundation (NRF) funded by the Ministry of Science and ICT (MSIT) (No. NRF-2014M3C9A3063541), and the Basic Science Research Program through the National Research Foundation of Korea (NRF) funded by the Ministry of Education (2020M3C9A5085604).

## Conflict of Interest

The authors declare that the research was conducted in the absence of any commercial or financial relationships that could be construed as a potential conflict of interest.

## Publisher's Note

All claims expressed in this article are solely those of the authors and do not necessarily represent those of their affiliated organizations, or those of the publisher, the editors and the reviewers. Any product that may be evaluated in this article, or claim that may be made by its manufacturer, is not guaranteed or endorsed by the publisher.
