# Corrigendum: MONTI: A Multi-Omics Non-Negative Tensor Decomposition Framework for Gene-Level Integrative Analysis

**DOI:** 10.3389/fgene.2021.778490

**Published:** 2021-10-25

**Authors:** Inuk Jung, Minsu Kim, Sungmin Rhee, Sangsoo Lim, Sun Kim

**Affiliations:** ^1^ Department of Computer Science and Engineering, Kyungpook National University, Daegu, South Korea; ^2^ Computing and Computational Sciences Directorate, Oak Ridge National Laboratory, Oak Ridge, TN, United States; ^3^ Department of Computer Science and Engineering, Seoul National University, Seoul, South Korea; ^4^ Interdisciplinary Program in Bioinformatics, Seoul National University, Seoul, South Korea

**Keywords:** feature selection, tensor decomposition, cancer, multi-omics, integrative analysis

There is an error in the **Funding statement**. The correct number for “the Basic Science Research Program through the National Research Foundation of Korea (NRF) funded by the Ministry of Education” is “2020M3C9A5085604.” Corrected statement is given below:

This research was supported by the Bio & Medical Technology Development Program of the National Research Foundation (NRF) funded by the Korean government (MSIT) (2019M3E5D3073365), the Collaborative Genome Program for Fostering New Post-Genome Industry of the National Research Foundation (NRF) funded by the Ministry of Science and ICT (MSIT) (No. NRF-2014M3C9A3063541), and the Basic Science Research Program through the National Research Foundation of Korea (NRF) funded by the Ministry of Education (2020M3C9A5085604).

The authors apologize for this error and state that this does not change the scientific conclusions of the article in any way. The original article has been updated.

